# Supplementary material for: Reactive Dicarbonyl Scavenging Effectively Reduces MPO-Mediated Oxidation of HDL and Restores PON1 Activity
Source: Nutrients. 2020 Jun 30;12(7):1937. doi: 10.3390/nu12071937 (PMC7400685; doi:10.3390/nu12071937)
Supplement: Supplementary file 1 [file nutrients-12-01937-s001.pdf]

Supplemental materials

Article

Reactive dicarbonyl scavenging effectively reduces MPO-mediated oxidation of HDL and restores PON1 activity

Jiansheng Huang[1], Patricia G. Yancey[1], Huan Tao[1], Mark S. Borja[2], Loren E. Smith[3], Valentina Kon[4], Sean S. Davies[5] and MacRae F. Linton[1,5]

1. Department of Medicine, Division of Cardiovascular Medicine, Atherosclerosis Research Unit, Vanderbilt University Medical Center, Nashville, TN 37232
2. Department of Chemistry & Biochemistry, California State University East Bay, CA
3. Department of Anesthesiology, Vanderbilt University Medical Center, Nashville, TN
4. Department of Pediatrics, Vanderbilt University Medical Center, Nashville, TN 37232
5. Department of Pharmacology, Vanderbilt University, Nashville, TN 37232

* Correspondence: macrae.linton@vumc.org

Running title: Dicarbonyl Scavengers Reduce MPO-Mediated Oxidation of HDL

Received: date; Accepted: date; Published: date

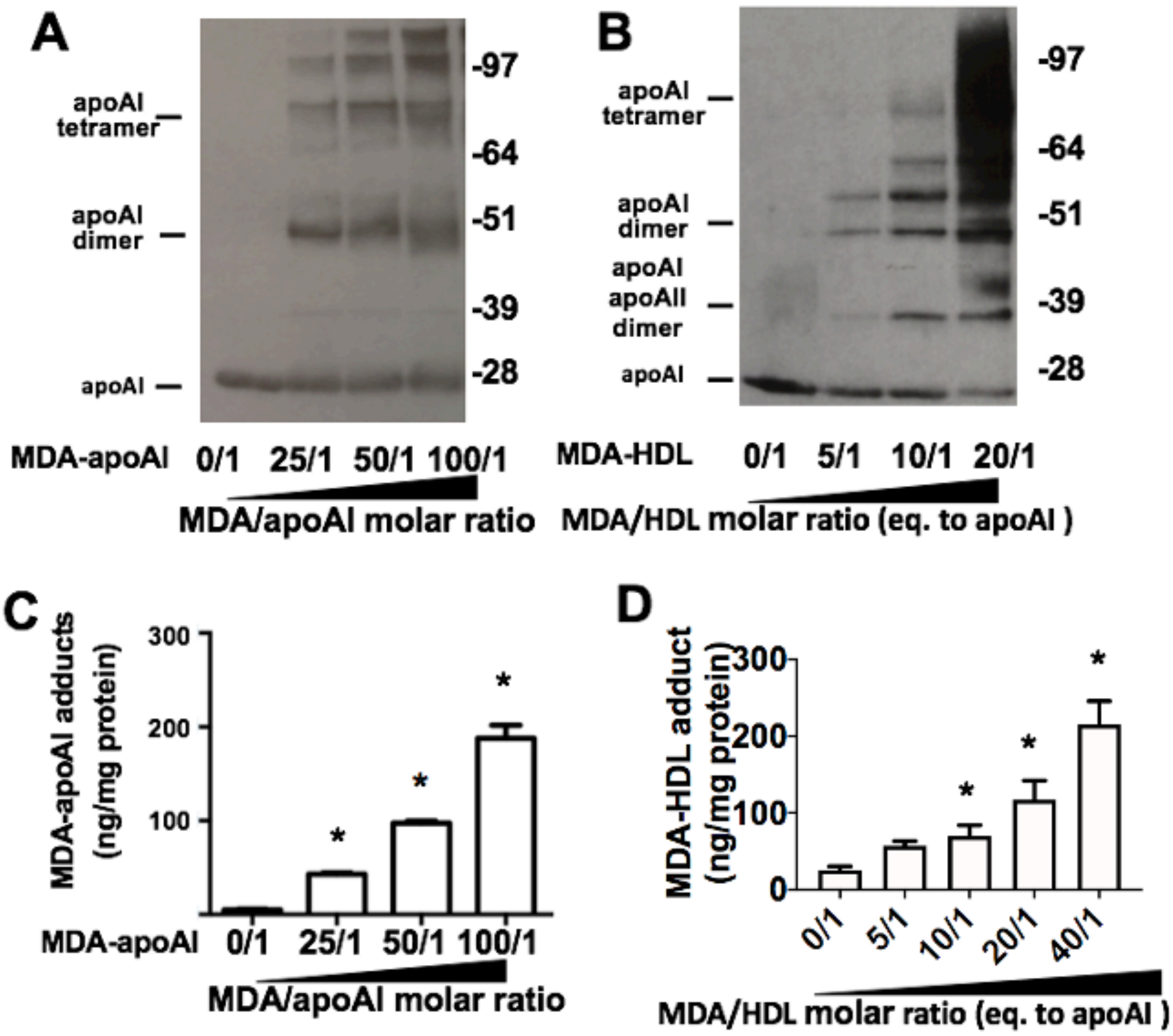

Supplemental Figure 1. Effects of MDA modification of apoAI or HDL on apoAI crosslinking. (A-D) ApoAI or HDL were modified with increasing concentrations of MDA. Briefly, 20 μL of 1 M HCl was added to 200 μL of maloncarbonyl bis-(dimethylacetal), and the mixture was incubated for 45 min at room temperature. The MDA concentration was determined by absorbance at 245 nm, using the coefficient factor 13, 700 M^{-1} cm^{-1}. ApoAI (1mg protein / mL) or HDL (10mg of protein /mL) and increasing doses of MDA (0, 0.125 mM, 0.25 mM, 0.5 mM, 1 mM) were incubated at 37 ^{o}C for 24 h in 50 mM sodium phosphate buffer (pH7.4) containing DTPA 100 μM. Reactions were initiated by adding MDA and stopped by dialysis of samples against PBS at 4 ^{o}C. The crosslinking of apoAI was determined in MDA modified apoAI (A) and HDL (B) by western blotting with anti-ApoAI antibody. Formation of MDA-apoAI adducts was measured in both MDA-modified apoAI (C) and MDA-modified HDL (D) by ELISA. Graphs represent data (mean ± SEM) of the experiments; *P <0.05 is indicated by comparing with apoAI or HDL group using one way ANOVA.

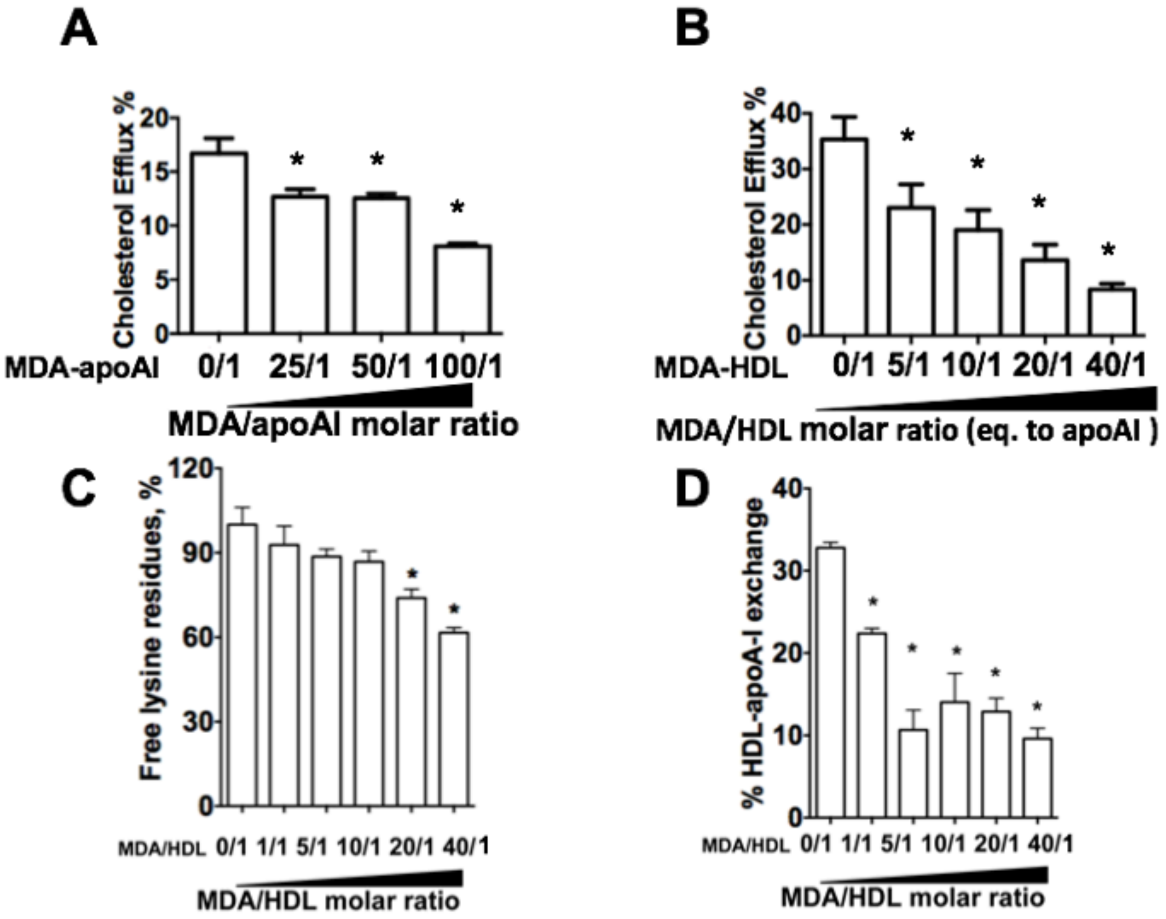

Supplemental Figure 2. Effects of MDA modification on cholesterol efflux capacity, apoAI lysine availability, and HDL-apoAI exchangeability. ApoAI (A) or HDL (B) were modified with increasing concentrations of MDA and the effects on cholesterol efflux capacity were determined. The availability of free lysines after MDA modification of HDL was measured using the OPA assay (C). The effects of MDA modification of HDL on apoAI exchangeability was measured by EPR (D). Graphs represent data (mean ± SEM) of the experiments; *P <0.05 is indicated by comparing with HDL only group using one way ANOVA.
